# Individual characteristics associated with the utilization of nursing care in the very old population: a cross-sectional study

**DOI:** 10.1186/s12877-022-03448-y

**Published:** 2022-09-20

**Authors:** Jaroslava Zimmermann

**Affiliations:** Cologne Center of Ethics, Rights, Economy, and Social Science of Health, University of Cologne, Albertus-Magnus-Platz, 50923 Cologne, Germany

**Keywords:** Community-dwelling, Nursing home, Frailty, Social factor, Advanced age

## Abstract

**Background:**

As older adults ≥80 years are often underrepresented in previous studies, little is known about their characteristics associated with the utilization of nursing care services. Therefore, this study examined individual (predisposing, enabling, and need) predictors of nursing care utilization in the very old population of North Rhine-Westphalia (NRW) in Germany.

**Methods:**

Data from a representative cross-sectional study included 1531 community-dwelling individuals and nursing home residents aged ≥80 years. Multinomial regression was applied to investigate the factors that explain the use of outpatient care services, day care, and/or private care (odpNCU), and inpatient nursing care (inpNCU).

**Results:**

Overall, 1083 (69.9%) participants did not use nursing care services (noNCU), 339 (21.9%) used outpatient, day, and/or private care, and 127 (8.2%) used inpatient nursing care. Compared to noNCU, odpNCU was associated with a higher likelihood of being older [odds ratio (OR) = 1.06, 95% confidence interval (CI): 1.01-1.11], having no partner (OR = 0.58, 95%CI: 0.37-0.91), experiencing higher functional (basic activities of daily living, OR = 0.02, 95%CI: 0.01-0.04; instrumental activities of daily living, OR = 0.18, 95%CI: 0.11-0.30) and cognitive disabilities (OR = 0.63, 95%CI: 0.44-0.89). Compared to noNCU, nursing home residents were more likely older (OR = 1.14, 95%CI: 1.07-1.22), had lower socioeconomic status (OR = 0.98, 95%CI: 0.97-1.00), were childless (OR = 3.83, 95%CI: 1.71-8.56) and without partners (OR = 0.43, 95%CI: 0.20-0.96), socially isolated (OR = 3.94, 95%CI: 2.06-7.55), were more likely to be lonely (OR = 2.94, 95%CI: 1.58-7.89), more functionally (basic activities of daily living, OR = 0.01, 95%CI: 0.00-0.03; instrumental activities of daily living, OR = 0.04, 95%CI: 0.02-0.09) and cognitively impaired (OR = 0.48, 95%CI: 0.31-0.74), but they were less likely to experience five or more chronic conditions (OR = 0.42, 95%CI: 0.20-0.88) and less likely to be physically pre-frail (OR = 0.24, 95%CI: 0.10-0.58) and frail (OR = 0.09, 95%CI: 0.03-0.27).

**Conclusions:**

Individual need factors dominated in explaining odpNCU, suggesting that the very old population in NRW may have equitable access to these services. As social structure, region, and social resources explain inpNCU, this type of care may be inequitably accessible.

## Background

In Germany, as in many European countries, an increase in the proportion of the population aged ≥80 years (further to very old adults) can be observed [1]. Despite the majority of very old adults living at home, the proportion of individuals in need of nursing care has continually increased in this age group over the last few years [2, 3]. Particularly after 2017, an increase approximately 20% was observed, which can be partly attributed to legislative changes, including the implementation of a new assessment instrument to define care dependency [2, 3]. For instance, the new definition allows not only persons with physical limitations (as previously defined) but also those with mental and cognitive limitations to be recognized as care-dependent. The increasing number of people in need of care constitutes a challenge for healthcare systems, and not only because of the growing health care costs. In Germany, the number of care-dependent individuals increased significantly faster than that of staffing levels in professional nursing care services [2, 3]. Identification of individual characteristics associated with nursing care utilization (NCU) can predict future requirements for nursing care services and enable to plan targeted interventions to enhance individual resources to decrease NCU.

In this study, NCU refers to the utilization of formal nursing care provided by outpatient (home) nursing care services, daycare, or inpatient nursing homes, and receiving informal care (e.g., provided by a relative or friend). Although very old adults are the most frequent nursing care users [4], few studies in Germany examine individual determinants of NCU in younger adults [5–7]. Therefore, the aim of the present study was to explore the individual characteristics associated with the NCU of the very old population in North Rhine-Westphalia (NRW), the most populated federal state of Germany. In NRW, the proportion of the very old population was 6.2% in 2017, the same as that in Germany [8]. According to a German long-term care insurance, 37.4% of the individuals aged 80 years and older were care-dependent in NRW (Germany, 36.5%), of which 71.5% (Germany, 69.6%) received home care (formal or informal) and 28.5% (Germany, 30.4%) were nursing home residents [4].

To identify relevant predictors, the behavioral model of health service use developed by Andersen [9] was adapted, which differentiates three types of determinants:

First, *predisposing factors* involve characteristics that increase the likelihood of NCU, such as demographic or social background. In most studies, older age [6, 10–16] and belonging to the ethnic majority [15, 17] were consistently reported as predisposing risk factors for home care or nursing home utilization. However, there are inconclusive findings regarding sex and social background. On the one hand, higher education level [5, 10] and female sex [5, 15, 16] was associated with NCU. However, other studies failed to identify any associations in this regard [6, 12, 14, 17]. A study in the United States found that older adults living in rural areas had a higher risk of nursing home admission [15].

Second, *enabling determinants* include the resources needed to use care services, such as social relationships, which may impede or facilitate NCU. There is an overwhelming consensus about the increased likelihood of using formal caregiving services for older adults who experience a lack of social support and loneliness [11, 14, 16, 18]. Cegri et al. [12] found that social vulnerability (assessed, for example, on the basis of family situation or social relations) significantly predicted admission to home care and nursing homes during the 8-year follow-up. Having children or regular contact with them [13, 15, 17], being married [15, 16], and living together with at least one other person in the same household [5, 6, 11–14] were identified as resources protecting oneself from NCU. The size of the social network was not associated with NCU [19].

Third, the *need for care* is the most important determinant as it relates to a person’s health status. Unsurprisingly, functional ability has been found to be a key factor. The majority of studies reported a higher need for assistance in performing basic activities of daily living (ADL) and/or instrumental activities of daily living (IADL) associated with NCU [5, 6, 10, 12–15]. Similarly, decreased cognitive function increased the risk of NCU [6, 11, 12, 14, 17]. Using different assessment tools, previous studies [18, 20–22] have consistently shown that physical frailty is associated with an increased use of home care services. Based on the Tilburg frailty indicator, Verver et al. [21] observed that, in addition to physical frailty, the combination of physical, social (e.g., living alone, low social support), and psychological frailty (e.g., problems with memory) contributed to a higher use of informal and home care. Cegri et al. [12] found that individuals who rated their health status as poor at the study baseline had a higher risk of admission to home care or nursing homes over the next 8 years. Kehusmaa et al. [14] demonstrated that frequent utilization of care services can contribute to an improvement in perceived health status over time. Other studies could not confirm an association between subjective health and NCU [6, 15, 17]. Similar inconclusive results were found regarding the association between multimorbidity and NCU [5, 12, 16].

Following Anderson [9], this study examined which predisposing, enabling, and need factors, contribute to the explanation of nursing care utilization among the very old population in NRW.

## Methods

### Study design

Data from the representative study “Quality of Life and Well-Being of the Very Old in North Rhine-Westphalia” (NRW80+) were collected between August 2017 and February 2018. The sampling procedure consisted of two steps. First, a random sample of 94 communities was selected from all NRW communities. Second, the registration offices of the selected communities provided a random sample of residents living in private households and institutions including individuals who had reached 80 years of age by July 31, 2017, and whose primary residence was registered in NRW (*N* = 48,137). Based on a priori power analysis and expected response rate of 20-25%, a gross sample size of 8040 individuals was determined. Individuals aged 85-89, 90 years and older, and men were oversampled to enable in-depth subgroup analyses. A total of 1863 computer-assisted personal interviews were realized which corresponds a response rate of 23.2%. These included 176 proxy interviews which were conducted when the target individuals were unable to participate owning to health impairment. In addition to the interviews, the interviewers assessed details about the living environment of the target individuals (e.g., place of residence, location) and interview situation (e.g., interruptions during the interview, other individuals being present). In all analyses, data weights were applied which were calculated based on the gross sample (to correct for community selection and oversampling). Moreover, data weights were calibrated using the known demographic characteristics of the very old population in NRW (e.g., age, sex, marital status, living in a nursing home, and region). Detailed information on methodical approach of NRW80+ has been published elsewhere [23, 24].

### Measures

#### Dependent variable

To define the utilization of nursing care among the participants, data from two different sources were used. First, the respondents provided information on the type of nursing care they were currently receiving. Initially, the participants specified whether they were receiving full inpatient nursing care (yes/no). Within NRW80+, receiving full inpatient nursing care was described as living in a nursing home, being care-dependent, and having access to 24-hour assistance and nursing care provided by the nursing staff. If this was not the case, participants were asked whether they were using outpatient nursing care services, day care in a nursing facility, and/or private (informal) care (e.g., provided by a relative or friend). Second, the interviewer’s assessment of living arrangements was additionally considered as we found several indicators that the self-reported type of nursing care received could not be plausible for a few respondents. These cases are described in greater detail below.

*No nursing care use (noNCU)* was attributed to persons who did not use any of the nursing care services (full inpatient, outpatient, day, or private care); at the same time, they lived in a typical private, multigenerational, or assisted living apartment/house or whose residential form was unknown.

Regardless of residential form, participants who reported using outpatient, day and/or private care were assigned to the *outpatient/day/private nursing care use (odpNCU)* group.

Finally, *inpatient nursing care use (inpNCU)* was given, when respondents lived in nursing homes, residential care groups, retirement homes, or senior residences according to the interviewer’s assessment, and they reported receiving full inpatient nursing care.

#### Independent variables

Based on previous findings, age (years), migration background (no/yes), sex, and socioeconomic status (SES) were included as *predisposing factors*. Using the International Standard Classification of Occupations 2008, defined by Ganzeboom [25], SES refers to the last occupational position of the respondent before retirement. The scale ranged from 16 (e.g., cleaners or helpers) to 90 (judges) [26]. In addition, community size considering four regional types was included; the operationalization was described in detail elsewhere [27].

*Enabling factors* included presence of children (no/yes), partnership status (in partnership vs. no partnership), time spent with others (never or rarely, sometime, often or very often), loneliness (never or sometimes, most of the time or always), and social isolation (no/yes). Following Huxbold and Engstler [28], individuals were defined as socially isolated if they could not name more than one person on their social network with whom they had contact at least once a week. Household members were counted as a part of the social network.

Finally, functional and cognitive impairment, multimorbidity, physical frailty, and subjective health status were considered *need factors*. Functional abilities were assessed using seven items referring to IADL and seven items related to ADL [29]. For IADL and ADL, the mean scores were calculated ranging from 0 (not possible without help) to 2 (no help needed). Based on the SF-8 [30], self-rated health status was measured using one item (very good, rather good, rather poor, or very poor). Multimorbidity was operationalized as the experience of five or more chronic conditions using self-reported medically treated diseases. For more information on the assessment of multimorbidity in NRW80+, see Brijoux et al. [31]. To determine cognitive impairment, the DemTect screening tool [32] was applied, defining at cut-off of eight and fewer points as dementia, and nine to 12 points as mild cognitive impairment [31]. Physical frailty was defined based on a study by Fried et al. [33] using the following four frailty criteria: exhaustion, unintentional weight loss, weakness, and low physical activity. Respondents meeting four or three criteria were defined as frail, those meeting two or one criterion were classified as pre-frail, and those meeting non-criteria were categorized as robust. Details of the operationalization of physical frailty within NRW80+ have been published elsewhere [27].

### Statistical analyses

Participants whose handgrip strength was not measured because the interviews were conducted with proxies (*N* = 176), and those who refused to perform the handgrip strength test (*N* = 110) were excluded from the analyses. The handgrip strength test score was required for the operationalization of weakness (physical frailty criterion). Similarly, respondents were excluded when they denied the cognition test DemTect (*N* = 46). The final sample included 1531 very old adults. Multiple imputations were applied to replace missing values. The majority of missing values were in cognition scores (15.2%) and SES (2.9%). Twenty imputed datasets were generated. The results of the original (non-imputed) dataset were only reported when they differed significantly from the imputed dataset. Multinomial regression was applied to analyze the (predisposing, enabling, and need) factors for the utilization of nursing care in very old adults. Statistical significance was set at *p* < 0.05. The variance inflation factors did not exceed the threshold of two for any of the included variables. IBM SPSS Statistics (Version 26) was used for all the analyses.

## Results

As noted before, the classification into the NCU groups was inconclusive in some cases. Few participants reported using no nursing care, although according to the interviewers, they lived in nursing homes (*N* = 3), residential care groups (*N* = 1), retirement homes (*N* = 21), or senior residences (*N* = 8). These participants were predominantly non-care-dependent (collected as a self-reported degree determining the need for long-term care according to the German long-term care insurance). Given that there are different hybrid forms of nursing care and housing options for older adults in Germany [34], which may have been difficult for interviewers to distinguish, self-reported information (no nursing care use) is prioritized in these cases. Additionally, two respondents were included in the odpNCU group, although they reported receiving full inpatient nursing care. According to the interviewers, these individuals lived in typical private houses or apartments. As mentioned above, a person receiving full inpatient nursing care must live in a nursing home that can be clearly distinguished from private housing during on-site interviews. Moreover, further available information indicates that these two individuals lived in a private household (according to registered data), and they did not move within the last 3 years (self-reported). Therefore, we proposed that these participants were more likely to receive outpatient or day nursing care.

In our study sample, 1083 (69.9%) very old adults did not use any nursing care; 339 (21,9%) used outpatient services, day, and/or private care. Inpatient nursing care was provided to 127 (8.2%) very old adults. For further details on the sample description, see Table 1.

**Table 1:** Description of study population differenciated by utilization of nursing care (*N* = 1549)

Variable	Total	NoNCU	OdpNCU	InpNCU
Total: weighted/unweighted	*1549/1531 (100%/100%)*	*1083/1051 (69.9%/68.6%)*	*339/381 (21.9%/24.8%)*	*127/100 (8.2%/6.5%)*
Age (years), mean (SD)	84.8 (4.0)	84.0 (3.4)	86.0 (4.1)	88.3 (5.0)
Sex				
male	575 (37.1%)	451 (41.6%)	107 (31.5%)	17 (13.5%)
female	974 (62.9%)	632 (58.4%)	233 (68.5%)	109 (86.5%)
Socioeconomic status^a^, mean (SD)	41.5 (20.7)	43.8 (20.9)	37.1 (20.0)	33.6 (16.7)
Migration background				
no	1189 (76.7%)	832 (76.8%)	263 (77.4%)	94 (74.6%)
yes	360 (23.3%)	251 (23.2%)	77 (22.6%)	32 (25.4%)
Community type				
5000 to 49,999	207 (13.4%)	139 (12.8%)	58 (17.1%)	10 (7.9%)
50,000 to 99,999	207 (13.4%)	137 (12.7%)	44 (12.9%)	26 (20.5%)
100,000 to 499,999	527 (34.0%)	363 (33.5%)	126 (37.1%)	38 (30.1%)
≥ 500,000	607 (39.2%)	443 (40.9%)	112 (32.9%)	52 (41.5%)
Children				
no	178 (11.5%)	127 (11.7%)	27 (8.0%)	24 (19.0%)
yes	1371 (88.5%)	956 (88.3%)	313 (92.0%)	102 (81.0%)
Partnership status				
no partnership	901 (58.2%)	553 (51.0%)	240 (70.8%)	108 (85.7%)
in a partnership	647 (41.8%)	530 (49.0%)	99 (29.2%)	18 (14.3%)
Social isolation				
no	1213 (78.4%)	865 (79.9%)	283 (83.4%)	65 (51.8%)
yes	335 (21.6%)	218 (20.1%)	56 (16.6%)	61 (48.2%)
Loneliness				
never/rarely	1471 (95.0%)	1051 (97.0%)	308 (90.9%)	112 (88.1%)
mostly/always	78 (5.0%)	32 (3.0%)	31 (9.1%)	15 (11.9%)
Time spent with others				
never/rarely	225 (14.5%)	150 (13.9%)	61 (18.0%)	14 (11.3%)
sometimes	393 (25.3%)	257 (23.7%)	98 (28.9%)	37 (29.6%)
often/very often	931 (60.1%)	676 (62.4%)	181 (53.1%)	75 (59.1%)
Self-rated health status, mean (SD) (1 = very poor; 4 = very good)	2.7 (0.8)	2.7 (0.7)	2.4 (0.8)	2.7 (0.8)
Basic activities of daily living, mean (SD) (0 = not possible without help; 2 = no help needed)	1.7 (0.4)	1.9 (0.2)	1.3 (0.5)	1.2 (0.6)
Instrumental activities of daily, living, mean (SD) (0 = not possible without help; 2 = no help needed)	1.5 (0.6)	1.8 (0.3)	1.0 (0.5)	0.7 (0.6)
≥5 chronical conditions				
no	1110 (71.7%)	829 (76.5%)	185 (54.7%)	96 (75.6%)
yes	438 (28.3%)	254 (23.5%)	154 (45.3%)	31 (24.4%)
Physical frailty				
robust	383 (24.8%)	347 (32.1%)	18 (5.3%)	18 (14.2%)
pre-frail	884 (57.0%)	624 (57.6%)	196 (57.7%)	65 (51.2%)
frail	281 (18.1%)	111 (10.3%)	126 (37.0%)	44 (34.6%)
Dementia (DemTect), mean (SD) (0 = dementia; 2 = no cognitive impairment)	1.6 (0.7)	1.8 (0.5)	1.4 (0.8)	0.9 (0.9)

Weighted data

*NoNCU* no nursing care use, *odpNCU* outpatient, day, and/or private nursing care use, *inpNCU* inpatient nursing care usem, *SD* standard deviation. When values differed among the imputed datasets, average values were reported. *p < 0.05; **p < 0.01; ***p < 0.001

^a^Based on Ganzeboom and Treiman [26], a value of 42 represents professions such as firefighters or aircraft engine mechanics; 44, general managers of restaurants and hotels (small enterprises); 37, market salespersons or bookbinders; and 34, heavy truck drivers or waiters/waitresses

As shown in Table 2, an increase in age was significantly associated with increased odds of odpNCU by 5.6% (95% confidence interval [CI]: 1.01-1.11) and inpNCU by 14.3% (95% CI: 1.07-1.22), both in relation to noNCU. An increase in SES by one unit (e.g., from senior officials to production department managers) was associated with a 1.7% (95% CI: 0.97-1.00) decrease in the odds of inpNCU. Very old adults who lived in communities with 5000 to 49,999 inhabitants (compared to 500,000 or more) had a 78.4% (95% CI: 0.08-0.59) lower likelihood of inpNCU. Not having children was associated with 3.83 (95% CI: 1.71-8.56) times higher odds of inpNCU. Having partners decreased the likelihood of odpNCU and inpNCU by 42.3% (95% CI: 0.37-0.91) and 56.7% (95% CI: 0.20-0.96), respectively. Social isolation was associated with 3.94 (95% CI: 2.06-7.55) times higher odds of inpNCU. Similarly, experiencing loneliness most of the time or always increased the likelihood of inpNCU by 2.94 times (95% CI: 1.58-7.89). A one-unit-increase in ADL (e.g., from not possible without help to possible with little help) was associated with decreased odds of odpNCU by 98.5% (95% CI: 0.01-0.04) and inpNCU by 99.0% (95% CI: 0.00-0.03). An increase in IADL declined the likelihood of odpNCU and inpNCU by 81.9% (95% CI: 0.11-0.30) and 95.6% (95% CI: 0.02-0.09), respectively. Experiencing five or more chronical conditions was linked to a 57.7% (95% CI: 0.20-0.88) decrease in the inpNCU. Pre-frailty (compared with robust) was associated with a 75.8% (95% CI: 0.10-0.58) decline in inpNCU, and frailty decreased the odds of inpNCU by 91.5% (95% CI: 0.03-0.27). A one-unit-increase in cognitive capacity (e.g., from dementia to mild cognitive impairment) reduced the likelihood of odpNCU by 37.5% (95% CI: 0.44-0.89) and inpNCU by 52.3% (95% CI: 0.31-0.74).

**Table 2:** Multinomial regression model of the associations of predisposing, enabling, and need factors with the utilization of nursing care (*N* = 1549)

	OdpNCU relative to noNCU			InpNCU relative to noNCU		
	*Odds ratio*	*95% CI*		*Odds ratio*	*95% CI*	
*Predisposing factors*						
Age	1.056*	1.005	1.109	1.143***	1.069	1.224
Sex (Ref: female)	1.132	0.721	1.776	0.509	0.235	1.104
Socioeconomic status	0.995	0.985	1.006	0.983*	0.967	1.000
Migration background (Ref: no)	0.665	0.422	1.050	0.928	0.477	1.808
Community type (Ref: ≥500,000)						
100,000 to 499,999^a^	1.498	0.959	2.340	0.563	0.282	1.124
50,000 to 99,999	1.178	0.647	2.146	1.253	0.548	2.867
5000 to 49,999	1.355	0.765	2.399	0.216**	0.079	0.592
*Enabling factors*						
Children (Ref: yes)^a^	0.713	0.390	1.302	3.827**	1.710	8.564
Partnership status (Ref: no partnership)^a^	0.577*	0.367	0.908	0.433*	0.195	0.962
Social isolation (Ref: no)	0.761	0.466	1.243	3.939***	2.056	7.546
Loneliness (Ref: never/rarely)^a^	2.004	0.950	4.227	2.942*	1.581	7.892
Time spent with others (Ref: often/very often)						
never/rarely	0.867	0.507	1.481	0.434	0.178	1.061
sometimes	0.939	0.600	1.471	0.800	0.405	1.581
*Need factors*						
Self-rated health status	1.045	0.799	1.366	1.469	0.973	2.218
Basic activities of daily living	0.015***	0.006	0.036	0.010***	0.004	0.029
Instrumental activities of daily living	0.181***	0.111	0.295	0.044***	0.020	0.094
≥ 5 chronic conditions (Ref: no)	1.203	0.789	1.833	0.423*	0.204	0.878
Physical frailty (Ref: robust)						
pre-frail^a^	1.436	0.779	2.648	0.242**	0.101	0.577
frail	1.136	0.528	2.445	0.085***	0.026	0.272
Dementia (DemTect)	0.625**	0.439	0.890	0.477**	0.309	0.738
*Pseudo R-squared (Nagelkerke)*^b^	0.695***					

Weighted data

*NoNCU* no nursing care use, *odpNCU* outpatient, day, and/or private nursing care use, *inpNCU* inpatient nursing care use, *CI* confidence interval, *ref* reference category

^*^p < 0.05

^**^p < 0.01

^***^p < 0.001

^a^The findings differed between the original and full imputed datasets. For more details, see “Results” section

^b^Calculated as the mean value from all imputed datasets

The following differences in the results of the original (non-imputed) dataset were found: not having children was significantly associated with decreased odds of odpNCU by 60.7% (95% CI: 0.19-0.84); relationship status was not associated with odpNCU; living in communities with 100,000 to 499,999 (compared to 500,000 or more) inhabitants significantly decreased inpNCU by 70.6% (95% CI: 0.13-0.69); and physical pre-frailty and loneliness were not associated with inpNCU.

Sensitivity analyses showed that individuals excluded from the analyses (*N* = 332) were older, more functionally impaired, had lower SES, lived more often in a nursing home, or used more frequently outpatient, day, and/or private nursing care compared to included participants (*N* = 1531),

## Discussion

In the study sample, 70% of the participants were identified as non-users of nursing care; 22% received outpatient, day, and/or private nursing care; and 8% were nursing home residents. Comparing the findings with official statistics of NRW [8], the proportion of noNCU in NRW80+ is slightly higher than that of individuals who did not require care according to the German Social Code XI in 2017 (63%). The proportions of odpNCU and inpNCU obtained in this study were slightly lower than those of the target population (27 and 11%, respectively) [8]. The differences might be interpreted by excluding proxy interviews conducted with particularly vulnerable very old adults. Nonetheless, the independent variables included in the analysis explained 70% of the variance in NCU among very old adults living in NRW. The results revealed that predisposing (age), enabling (partnership status), and need (functional and cognitive impairment) factors predicted the odpNCU. Several predisposing (age, SES, community size), enabling (presence of children, partnership status, social isolation, and loneliness), and need (functional and cognitive impairment, physical frailty, and multimorbidity) factors were significantly associated with inpNCU.

Consistent with previous studies on predictors of home care use [12, 16] and nursing home admissions [6, 11, 12, 15], the predisposing factor for increasing age was associated with odpNCU and inpNCU. The increasing nursing care needs with age might be interpreted as a consequence of age-associated decline in physiological functions, accompanied by increasing functional and cognitive impairments or increased risk for adverse health outcomes [35, 22]. In the present study, very old adults with a higher SES had a lower likelihood of inpNCU, in contrast to the findings of Yu et al. [10] and Steinbeisser et al. [5]. As the majority of previous studies considering education level as an indicator of social class found no relationship with inpNCU [5, 6, 10], this finding might indicate that the use of the last occupation before retirement [25] could be a more appropriate indicator of social status than educational level. Another explanation for these inconsistencies might be related to the older age of the study participants, suggesting an increase in the socioeconomic gradient in health over time [36, 37]. There is evidence that individuals with a higher SES experience better health status over their lifespan [36–38], which may result in a lower likelihood of NCU [39]. Furthermore, inconsistent with a study conducted in the United States [15], there was a lower likelihood of inpNCU among very old adults living in communities with fewer than 50,000 inhabitants. Considering the findings of Kuppler and Wagner [40], lower inpNCU in smaller communities might be attributed to lower inpatient nursing care supply in these areas of NRW. Another perspective provided in the study by Zimmermann et al. [27] reported a lower likelihood of physical frailty among very old adults living in small communities of NRW, which could lead to a lower demand for inpNCU.

Considering the enabling factors, similar to previous findings [15, 17], having children decreased the likelihood of inpNCU. Similarly, partnership reduced the demand for odpNCU [10, 12, 16] and inpNCU [6, 11, 15]. Moreover, social isolation and loneliness increased the likelihood of inpNCU, which is in accordance with the research literature [5, 6, 11, 12]. On one hand, these findings may indicate that the lack of close social connections (such as the absence of children or partners) and loneliness may facilitate the use of inpatient nursing care services. On the other hand, admission to long-term care facilities might lead to the loss of existing social networks, and consequently, loneliness [41]. Thus, the results indicate that the prevention of social isolation and loneliness, recognized as indicators of social frailty [18, 21], has not been sufficiently established in residential care facilities in NRW.

The majority of individual need characteristics played an important role in explaining the NCU of the very old population. In line with previous studies, participants with higher impairments in ADL and IADL had an increased likelihood of odpNCU [12–14] and inpNCU [5, 6, 12, 15]. Similarly, lower cognitive function was associated with an increased demand for odpNCU and inpNCU [6, 11, 12, 14]. In contrast to previous studies [5, 7, 12, 42], physical pre-frailty, frailty, and multimorbidity were lower by the inpNCU than by the noNCU. According to Andersen [9], protective effect of inpNCU on multimorbidity and physical frailty might be explained in terms of effective access to inpatient nursing care services in NRW, when the use of the services contributes to an improvement in physical health status. However, this effect could also be related to a bias in the analysis sample due to the excluded interviews.

The present study was the first to investigate the relationship between individual characteristics and different types of NCU using representative data for the very old population in NRW. Wagner et al. [43] found that respondent characteristics (age, sex, living in a private household or nursing homes) were not associated with the contact, cooperation, and response rate in NRW80+. Therefore, these findings can be assumed to be generalizable to the target population. For the definition of NCU, self-reported information and information from interviewers were considered to identify NCU status as accurately as possible. In addition to the sociodemographic and health characteristics included in previous German studies [5–7], social predictors of NCU (such as isolation or loneliness) were included. However, this study had some limitations. First, for the definition of physical frailty, four instead of the five criteria recommended by Fried et al. [33] were used because walking speed was not assessed in NRW80+. The exclusion of this criterion was previously found to have the smallest impact on the prediction of adverse health outcomes [44]. Second, the proxy interviews had to be excluded from the analyses because of the absence of measurement of the handgrip strength of the target individuals. Multiple imputations were performed to replace missing values to minimize possible bias. Third, owning to the analysis of imputed data, it was not possible to consider clusters of individuals within communities. Nevertheless, community selection in the study sample and regional characteristics (such as size or population density of communities) were adjusted through data weighting. Fourth, using multinomial regression, the assumption of independence between category memberships of the dependent variable might have been violated in the case of odpNCU and inpNCU. Finally, because cross-sectional data were used for the analysis, no conclusions about causal relationships could be derived.

## Conclusion

Applying Andersen’s behavioral model [9], the dominance of individual need factors in explaining odpNCU suggests that very old adults in NRW have equitable access to this type of nursing services, based on actual care demands. Instead, access to inpNCU can be characterized as inequitable because social structure, region, and enabling resources determine who receives nursing care [9]. The findings suggested that the inpNCU of very old adults might compensate for missing social resources (i.e., closest relatives as potential informal caregivers). Because evidence shows that social frailty predicts physical frailty, disability, and mortality [45–48], appropriate interventions to support the social inclusion of very old adults living in inpatient nursing care settings are required. In addition, measures to enhance the social participation of very old adults in communities could help reduce the risk of nursing home admission. Finally, there might be an indication of an inequitable supply of inpatient nursing care services in smaller NRW communities. Further studies are required to investigate this in more detail. At the community and federal state levels, the availability of nursing care services for very old adults, particularly in rural areas, should be ensured.

## Data Availability

The dataset generated and/or analyzed during the current study is available in the GESIS – Leibniz Institute for the Social Sciences data repository, 10.4232/1.13527. Additionally, the datasets used and/or analyzed during the current study available from the corresponding author on reasonable request.

## Abbreviations

ADL: Basic activities of daily living
CI: Confidence interval
IADL: Instrumental activities of daily living
inpNCU: Inpatient nursing care use
NCU: Nursing care utilization
noNCU: No nursing care use
NRW: North Rhine-Westphalia
NRW80 +: Representative study “Quality of Life and Well-Being of the Very Old in North Rhine-Westphalia”
odpNCU: Outpatient, day, and/or private nursing care use
SD: Standard deviation
SES: Socioeconomic status
